# Identification key to Nephtyidae (Annelida) of the Black Sea

**DOI:** 10.3897/zookeys.908.38203

**Published:** 2020-02-03

**Authors:** Nataliya Yu. Dnestrovskaya

**Affiliations:** 1 Faculty of Biology, Lomonosov Moscow State University, Moscow, 119234 Russia Lomonosov Moscow State University Moscow Russia

**Keywords:** East Atlantic, *
Inermonephtys
*, *
Micronephthys
*, *
Nephtys
*, Polychaeta

## Abstract

Currently, nine species of Nephtyidae (Annelida) are known from the Black Sea. A new user-friendly identification key is presented with a brief description for each species based on type material and recently collected specimens from the Black Sea.

## Introduction

The first data on Nephtyidae of the Black Sea were given by Bobretsky (1870), who listed a single species, *Nephtys hombergii* Savigny in Lamarck, 1818. At present, nine species of Nephtyidae are known from the Black Sea: *Inermonephtys foretmontardoi* Ravara, Cunha & Pleijel, 2010; *Micronephthys longicornis* (Perejaslavtseva, 1891); *Nephtys caeca* (Fabricius, 1780); *N. ciliata* (Müller, 1789); *N. cirrosa* Ehlers, 1868; *N. hombergii*; *N. hystricis* McIntosh, 1900; *N. longosetosa* Örsted, 1842; and *N. paradoxa* Malm, 1874 (Şahin and Çinar 2012; Çinar et al. 2014; Dnestrovskaya and Jirkov 2019).

The Black Sea nephtyid polychaetes are small to medium-sized bristle worms. The largest species, *N. caeca*, may reach a length of up to 250 mm (Rainer 1991), and the smallest (*M. longicornis*) up to 11 mm (San Martin 1982). Most collected worms are usually 20–50 mm long. In the Black Sea they can be found at depths of 0–600 m, and even deeper near the Bosphorus (Çinar et al. 2014), in a wide variety of substrates, but especially in soft sediments. Most nephtyids are considered to be actively burrowing carnivores that use muscular and rapidly everting pharynges with unhinged teeth to capture and crush prey. These worms feed on mollusks, crustaceans, and other polychaetes, which may include smaller conspecifics. *Nephtys hombergii* may switch to an omnivorous diet in certain habitats and when population density becomes high (Schubert and Reise 1986; Jumars et al. 2015; Ravara et al. 2017).

The illustrated key provided herein allows for the identification of Nephtyidae species reported from the Black Sea and Sea of Azov. This key is based mainly on external morphological characters, which are best viewed using a stereomicroscope. Staining with methylene blue makes all morphological characters more visible. No slide preparation or a compound microscope is needed. Each species of Nephtyidae is provided with a brief description and distribution. The names of provinces are given according to Briggs and Bowen (2012) and Golikov et al. (1990).

**Abbreviations**: KHB MSU = Department of Hydrobiology Lomonosov Moscow State University, Moscow, Russia; IO RAS = P.P. Shirshov Institute of Oceanology Russian Academy of Sciences, Moscow, Russia, IAZ SSC RAS = Institute of Arid Zones of Southern Scientific Center of the Russian Academy of Sciences, Rostov-on-Don, Russia; ZIN = Zoological Institute of Russian Academy of Sciences, St. Petersburg, Russia; IMBU = Institute of Marine Biology of the NAS of Ukraine, Odessa, Ukraine; IMBR = The A.O.Kovalevsky Institute of Marine Biological Research of RAS, Sevastopol, Russia; MCZ = Museum of Comparative Zoology of Harvard University, Cambridge, MA, United States; NHMUK = Natural History Museum, London, United Kingdom; NHC = Natural History Collections of University Museum of Bergen, Bergen, Norway. Abbreviations with numbers denote the chaetiger, i.e. C3 means the third chaetiger. All features used in the couplets are shown in the figures nearby.

## Materials and methods

**Examined type material**: *N. caeca*: NHMUK MO06 1847.9.15.10, paratype; *N. cirrosa*: MCZ IZ ANNa-1242, holotype; *N. hombergii*, NHMUK MO10 1863.9.19.12, holotype, NHMUK AN01 1921.5.1.810–813, NHMUK AN01 1921.5.1.814–815, paratypes; *N. hystricis*, NHMUK AN01 1921.5.1.767, NHMUK AN01 1921.5.1.768, NHMUK AN01 1921.5.1.782–783, NHMUK AN01 1921.5.1.769–770, NHMUK AN01 1921.5.1.784–790, NHMUK AN01 1921.5.1.781, NHMUK AN01 1921.5.1.765–766, NHMUK AN01 1921.5.1.791–795, NHMUK AN01 1921.5.1.771–780, paratypes.

**Additional material**: over 200 specimens from the Black Sea were also examined: *M. longicornis* (KHB MSU, IAZ SSC RAS, IO RAS); *N. cirrosa* (ZIN); *N. hombergii* (KHB MSU, NHMUK).

Four species, *N. caeca* (KHB MSU, APEM), *N. ciliata* (KHB MSU, NHC), *N. longosetosa* (KHB MSU, APEM), and *N. paradoxa* (KHB MSU, NHC, NHMUK), were described from material collected from the North, Norwegian, and Barents Seas (over 350 specimens).

Almost all samples were first fixed in 10% formaldehyde and then transferred to 70% ethanol (24 specimens of *M. longicornis* were fixed directly in 70% ethanol). Specimens were stained with methylene blue (water solution) and examined using stereomicroscopy. Pharynx characters were studied on worms with a fully everted pharynx. Photographs were taken using a Carton DSZT70 stereomicroscope equipped with a MDC 320 Microscope Digital Camera. Line drawings were prepared by tracing stereomicroscope photographs in CorelDRAW. To examine the ultrastructure of chaetae, some chaetigers were dissected, critical point dried, coated with 25 nm Au-Pd and observed with a Camscan S-2 Cambridge Scanning Electron Microscope (SEM).

**Figure 1.**
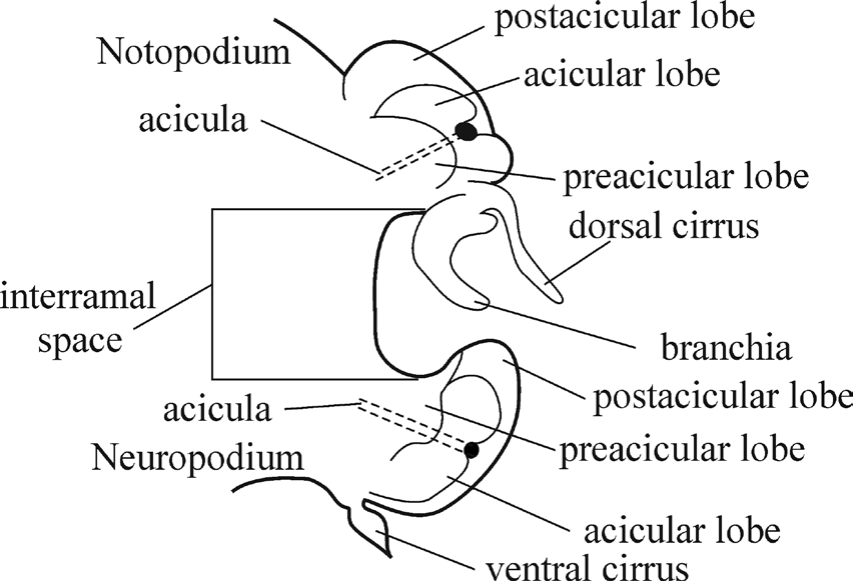
Explanation of main parapodia terminology.

**Figure 2.**
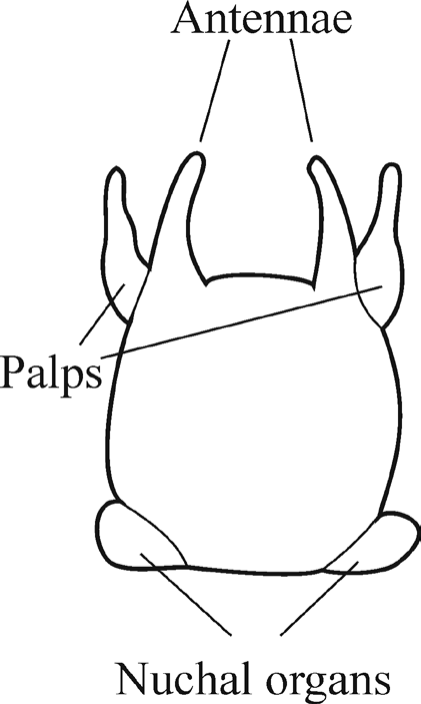
Explanation of prostomium terminology.

**Figure 3.**
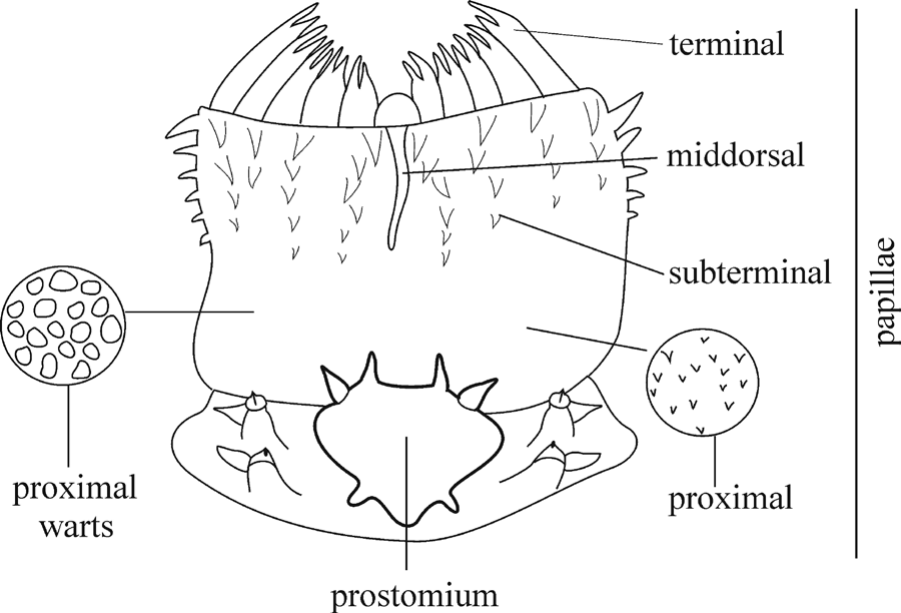
Explanation of main terminology of pharynx.

**Figure 4.**
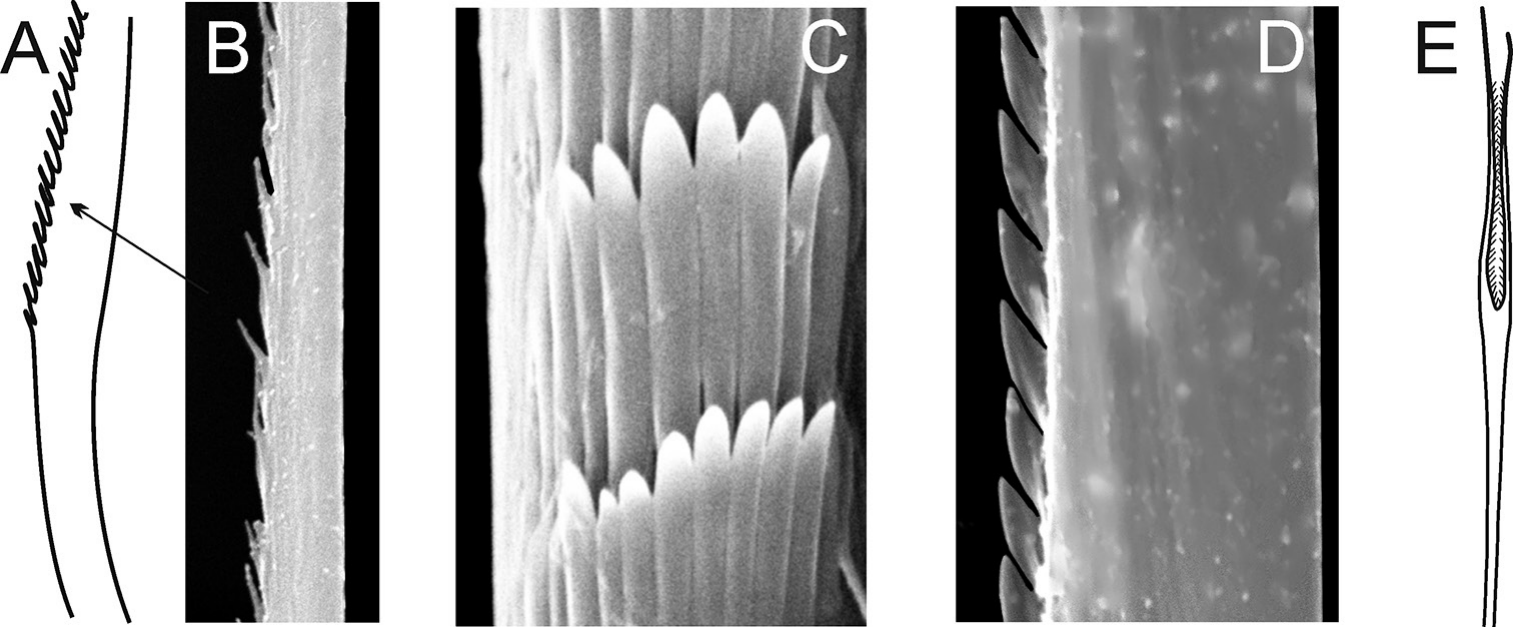
Explanation of main chaetae terminology (all chaetae of postacicular rows) **A, B** geniculate chaeta of *Nephtys cirrosa***C** spinose chaeta of *Nephtys ciliata*, frontal view **D** serrate chaeta of *Nephtys hombergii*, lateral view **E** lyrate chaeta of *Inermonephtys foretmontardoi* (after Ravara et al. 2010).

All the descriptions and drawings are original except for that of *I. foretmontardoi* (after Ravara et al. 2010). They were made without preparing slides as a cover glass deforms parapodial structure. All parapodia are shown in anterior view, unless otherwise stated. The terminology used in the key is given in Figures 1–4.

## Results

### Remarks on the key

Nephtyids are rather similar in their morphology and often difficult to distinguish. The most used taxonomic characters to separate the species are: parapodial morphology, branchiae shape, number of branchiferous chaetigers, ornamentation of the chaetae (only visible under a compound microscope), and pharynx structure. The number of the most anterior chaetiger with developed postacicular lobes was also included in descriptions, as this is an important systematic character (Dnestrovskaya 2013). Not all characters are developed in juveniles, and it is not always possible to identify fragmented animals without specialized training.

All parapodia in Nephtyidae are biramous. Both noto- and neuropodia consist of acicular, pre- and postacicular lobes, and dorsal (notopodial) and ventral (neuropodial) cirri. In *Nephtys* and *Micronephthys* species, the acicular lobes are supported by one acicula and may be conical, rounded, or bilobed (Fig. 1). In *I. foretmontardoi*, the anteriormost parapodia have up to five aciculae in the neuropodia and four in the notopodia. The number of aciculae decreases gradually towards the posterior end of the body. Single aciculae of posterior parapodia have curved tips. Smaller specimens of *Inermonephtys* have a lower number of aciculae per parapodium (after Ravara et al. 2010). Shape of parapodial lobes varies along the body, so the user should be sure of examining the parapodia from the chaetiger recommended in the key or key drawings. All morphological details of the parapodia can usually be seen under the stereomicroscope. Several undamaged parapodia from both sides of the worm should be examined.

The branchiae are inserted in the parapodia below the dorsal cirri and may be involute or recurved. They may be slender, digitiform, foliaceous, or rounded-fleshy. A small papilla may be present at the base of the branchiae under the notopodial cirrus. The shape and proportions of branchiae vary along the body, so they should be examined on the chaetigers that are recommended in the key. The chaetiger on which the branchiae begin should be checked on both sides of the worm.

The prostomium is subquadrangular to subpentagonal (shape depends on whether the proboscis is everted or not). A pair of conical antennae is present in the anterior corners of the prostomium (absent in *Inermonephtys*). A pair of palps is inserted ventrolaterally. A pair of nuchal organs is located dorsolaterally on the posterior margin of the prostomium (Fig. 2).

The pharynx is a large eversible muscular proboscis, usually covered with soft papillae located in different areas that can be seen when pharynx is everted or dissected (Fig. 3). All pharyngeal papillae are absent in *Inermonephtys*. In *Micronephthys* and *Nephtys* the anterior margin of the pharynx is surrounded by 18–20 bifid terminal papillae separated dorsally and ventrally by gaps; each gap may bear a single conical papilla. The subterminal region has 14 to 22 longitudinal rows of conical to digitiform papillae decreasing in size towards the base of the pharynx. A single longer subterminal papilla may be present middorsally and midventrally. The proximal surface may be smooth or covered with small warts (flat outgrowths) or small papillae (conical or rounded) that slightly rise above the surface. Pharynx dissection is not always necessary but may be useful to confirm identifications.

Examining several specimens rather than a single individual is strongly recommended for identification. Staining with methylene blue (but not methyl blue!) will significantly highlight morphological characters of all structures.

## Discussion

*Nephtys caeca*, *N. ciliata*, *N. longosetosa*, and *N. paradoxa* were absent not only in our collection from the Black Sea, but also in the collections of other museums where the Black Sea’s fauna traditionally was studied (ZIN; IMBR, Dr E. Lisitskaya pers. comm.; IMBU, Dr O. Bondarenko pers. comm.); they are absent in the keys by Vinogradov and Losovskaya (1968).

The mention of these species is based on identification by Rullier (1963) from the region near the Bosphorus. In later articles, Marinov (1977), Kiseleva (2004), Şahin and Çinar (2012), and Çinar et al. (2014) all referred to the same samples.

Perejaslavtseva (1891: 236) wrote: “Steamerships that constantly are coming from Constantinople and the Mediterranean, could bring to the Black Sea the specimens of the strait Bosphorus fauna”, and recent researchers agree with her (Ivanov and Belokopytov 2011). However, I believe that the ranges of *N. caeca*, *N. ciliata*, *N. longosetosa*, and *N. paradoxa* are too widely circumscribed. These species are probably absent from the Black Sea fauna and some may even be absent from the Mediterranean; at least their presence in these faunas needs confirmation. Close investigation of some other species with wide distributions (including the Arctic Ocean and Mediterranean Sea) has shown that in reality they are species complexes. For example, Jirkov’s (2018) recent revision of *Thelepus cincinnatus* (Fabricius, 1780) (Terebellidae) resulted in four different species: one from the deep Arctic, a second arcto-boreal, a third boreal-Mediterranean, and a fourth Mediterranean species.

The Nephtyidae of the Black Sea could be divided into two groups by the presence of different types of chaetae in the postacicular rows. The north-boreal species (*N. caeca*, *N. ciliata*, *N. longosetosa*, and *N. paradoxa*) have spinose chaetae, while south boreal-Lusitanian species (*N. hombergii*, and *N. hystricis*) have serrate chaetae with only single lateral rows of spines along one side of the chaeta (Dnestrovskaya and Jirkov 2011). *Nephtys cirrosa* has specific geniculate chaetae, *M. longicornis* has dentate chaetae and lyrate chaetae with unequal rami whereas *I. foretmontardoi* has lyrate chaetae with subequal rami (Ravara et al. 2010) (Fig. 4). Despite the shape of spines in postacicular chaetae only being visible under a compound microscope, they were added in descriptions as a supplementary character.

No key is complete and perfect. The key given below should be used with caution and confirmed with descriptions of the species concerned.

### List of Nephtyidae reported from the Black Sea

***Inermonephtys*** Fauchald, 1968

*Inermonephtys foretmontardoi* Ravara, Cunha & Pleijel, 2010

***Micronephthys*** Friedrich, 1939

*Micronephthys longicornis* (Perejaslavtseva, 1891)

***Nephtys*** Cuvier, 1817

*Nephtys caeca* (Fabricius, 1780)

*Nephtys ciliata* (O.F. Müller, 1789)

*Nephtys cirrosa* Ehlers, 1868

*Nephtys hombergii* Savigny in Lamarck, 1818

*Nephtys hystricis* McIntosh, 1900;

*Nephtys longosetosa* Örsted, 1842

*Nephtys paradoxa* Malm, 1874

### Key to nephtyid species from the Black Sea

**Table:** 

	1	Branchiae absent, up to 11 mm long (usually shorter), no more than 50 chaetigers (usually 30–40)	***Micronephthys longicornis***
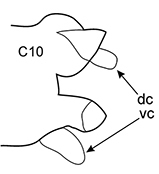	Small worms with body length up to 11 mm (San Martin 1982), up to 50 chaetigers (Laborda 2004); for Black Sea, up to 7.7 mm, up to 38 chaetigers (Dnestrovskaya and Jirkov 2019). Antennae long (near 2/3 length of prostomium) and digitiform, with swollen tips. One or two pairs (coalesced) of eyespots of irregular form visible dorsally on C3 or nearby. Branchiae absent. Parapodial preacicular and postacicular lobes rudimentary; acicular lobes conical. Notopodia of C1 with dentate chaetae. Lyrate chaetae with unequal rami from C3 in postacicular rows. Pharynx with up to 9 subterminal papillae per row, single middorsal and midventral subterminal papillae absent, proximal region smooth. Upper sublittoral. Lusitanian, within Mediterranean 3.6–7 m (Banse 1959; San Martin 1982; Laborda 2004; Ravara et al. 2010); in Black Sea much deeper, up to 47.4 m (Dnestrovskaya and Jirkov 2019).		
	–	Branchiae present, usually over several tens of chaetigers; up to 200 chaetigers or more and may be over 200 mm long in adults (in juveniles number of chaetigers may be low, but just before pygidium there is growth zone with numerous developing chaetigers)	**2**
	2(1)	Branchiae of middle parapodia curved inward	***Inermonephtys foretmontardoi***
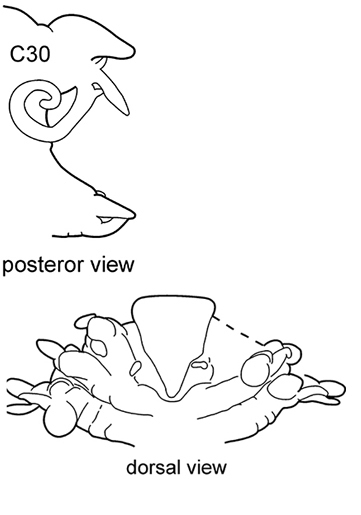	Body length more than 28.8 mm, more than 57 chaetigers (type specimen incomplete). Branchiae from C4, curved inward, thin and long, moderately ciliated, with conspicuous conical basal projections. Antennae and pharyngeal papillae absent. Nuchal organs cirriform. Preacicular lobes low, rounded. Acicular lobes rounded in anterior parapodia, conical in middle, acutely pointed in posterior. Postacicular lobes distinctly longer than acicular lobes, rounded in anterior notopodia and leaf-like in middle; always slender in neuropodia. Dorsal cirri conical in anterior parapodia, slender and cirriform in middle ones. Ventral cirri conical, as long as neuropodial postacicular lobes. Lyrate chaetae with subequal rami in postacicular rows. Anteriormost parapodia with up to 5 aciculae in neuropodia and 4 in notopodia. Pharynx smooth, without papillae (after Ravara et al. 2010). Lusitanian, shelf depths (Foret-Montardo 1969; Laborda 2004); Black Sea (Çinar et al. 2014).		
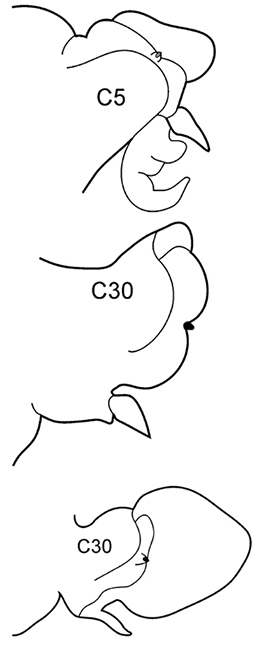	–	Branchiae of middle parapodia curved outward	*** Nephtys *** **3**
	3(2)	Neuropodial postacicular lobes of middle chaetigers (after C30) almost equal or shorter than acicular lobes	**4**
	–	Neuropodial postacicular lobes of middle chaetigers (after C30) distinctly longer than acicular lobes	**6**
	4(3)	In middle part of body (after C20) acicular lobes distinctly bilobed	***N. ciliata***
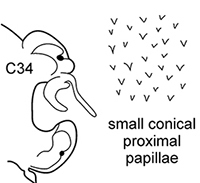	Body length up to 170 mm, up to 94 chaetigers. Branchiae from C8–C12 (rarely from C7), longer than dorsal cirri to C45–C55; decreasing in size to small knob posteriorly (shorter than dorsal cirrus), and then completely absent. Dorsal cirri of middle chaetigers long and cirriform. Notopodial preacicular lobes rudimentary, neuropodial preacicular lobes low, but distinct, in anterior and middle chaetigers surrounding acicular lobes interramally. Acicular lobes bilobed in anterior and middle region, rounded in posterior chaetigers. Postacicular lobes from C2 in neuropodia, C3 in notopodia. Notopodial postacicular lobes shorter or subequal in length to acicular lobes, neuropodial postacicular lobes subequal in length to, or slightly longer than, acicular lobes. Spinose chaetae in postacicular rows. Pharynx with long middorsal subterminal papilla, up to 7 subterminal papillae per row, proximal region covered with small conical papillae. Arcto-boreal, mainly lower shelf; reported from Black Sea near Bosphorus (Rullier 1963), but these records require confirmation.		
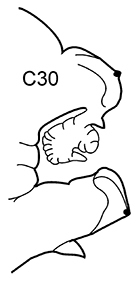	–	In middle part of body (after C20) acicular lobes rounded or conical	**5**
	5(4)	Preacicular lobes rounded and rudimentary. Branchiae from C5–C20, very minute at first, in the middle chaetigers often (not always) more or less foliaceous	***N. paradoxa***
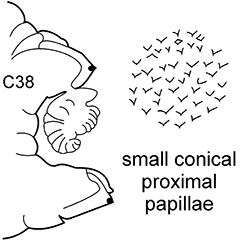	Body length up to 200 mm, up to 150 chaetigers (Rainer 1991). Branchiae from C5–C20 (usually C10–C11), minute at first, gradually increasing in size to C25–C27, in middle chaetigers often (but not always) more or less foliaceous and rounded-fleshy, their thickness not varying from center to edges. Branchiae decreased from C40–C45, absent from C50–C60 (last 25 or 30 chaetigers according to Hartman 1950). Parapodial preacicular lobes rudimentary or poorly developed; surrounding acicular lobes interramally in neuropodia of anterior and middle chaetigers. Anterior notopodial acicular lobes may be slightly bilobed, posteriorly rounded-conical. Neuropodial acicular lobes rounded-conical, posteriorly conical. Postacicular lobes from C2 in neuropodia, C3 in notopodia; in anterior and middle parapodia subequal in length to or slightly longer than acicular lobes, posteriorly shorter than acicular lobes. Spinose chaetae in postacicular rows. Pharynx with short middorsal subterminal papilla, up to 6 subterminal papillae per row; in large worms proximal region of pharynx sometimes covered with small conical papillae. Arcto-boreal, lower shelf depths. Reported from Black Sea near Bosphorus (Rullier 1963), but these records require confirmation.		
	–	Preacicular lobes of middle chaetigers (between C15 and C45) distinctly bilobed. Branchiae from C6–C7	***N. hystricis***
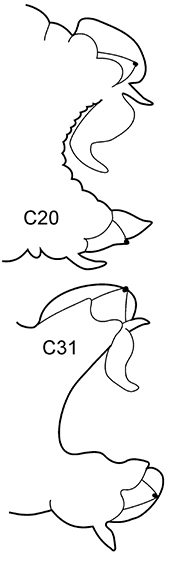	Body length up to 50 mm (Laborda 2004), up to 75 chaetigers (Hartmann-Schröder, 1996). Branchiae usually from C6–C7, rarely C5 (C7–C9 after Laborda 2004), maximum development in C25–C40 (Laborda 2004; Rainer 1990), then greatly reduced, absent in 18–20 posterior chaetigers (15–18 after Rainer 1990, 11–21 after Ravara et al. 2010). Preacicular lobes shorter than acicular lobes, rounded in anterior and posterior chaetigers, bilobed in middle (C15–C50), with more prominent interramal parts in C20–C30 (Laborda 2004; Rainer 1990). Acicular lobes conical, posteriorly (after C50) acutely conical. Postacicular lobes from C3 in neuropodia, C5 in notopodia. Notopodial postacicular lobes broadly rounded; in anterior chaetigers dorsally and distally longer than acicular lobes (up to C20); in middle chaetigers obliquely rounded, dorsally longer and distally equal to acicular lobes (C30–C40); posteriorly reduced and shorter than acicular lobes in both dimensions. Neuropodial postacicular lobes broadly rounded; distally longer than acicular lobes in anterior chaetigers (up to C20), equal to acicular lobes in middle chaetigers, decreasing in size posteriorly. Serrate chaetae in postacicular rows. Pharynx with long middorsal subterminal papilla, up to 6 subterminal papillae per row, proximal region smooth. Boreal, shelf depths (mainly lower shelf).		
	6(3)	In middle chaetigers: preacicular lobes distinctly bilobed, branchiae with visible basal outgrows under dorsal cirri (arrow a)	***N. hombergii***
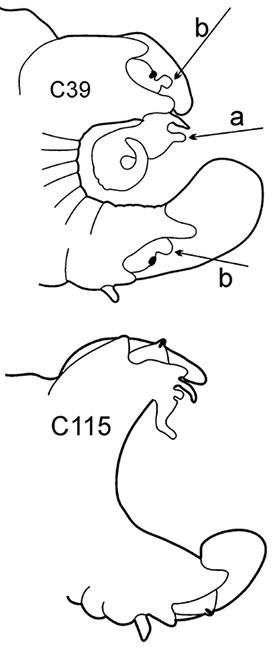	Body length up to 200 mm up to 200 chaetigers (Fauvel 1923). Parapodia fully developed from C25–C30. Branchiae from C4–C5 (rarely from C6) to near posterior end, with distinct basal outgrows under dorsal cirri. Preacicular lobes rounded in utmost anterior chaetigers; bilobed from C15–C20 to near posterior end, with equal parts in notopodia and larger interramal parts in neuropodia. Acicular lobes with interramal outgrows (arrows b) from C5 to C60–C65, best seen in C25–C40. Postacicular lobes from C3 in neuropodia, C4 in notopodia. Neuropodial postacicular lobes broadly rounded, more than twice longer than acicular lobes in anterior chaetigers (C4–C30); more than three times longer in middle chaetigers (C30–C80). Notopodial postacicular lobes up to twice as long as acicular lobes (from C5 till C60–C65), posteriorly equal to acicular lobes. Serrate chaetae in postacicular rows. Pharynx with long middorsal subterminal papilla, proximal region smooth, up to 6 papillae per row (Rainer 1991; Ravara et al. 2010). Lusitanian, low boreal, from intertidal to continental shelf depths, (Rainer 1991), in Black Sea up to 110 m depth (material from ZIN).		
	**Remark**: interramal outgrows in acicular lobes may be poorly expressed in specimens from Azov Sea and Kerch Strait.			
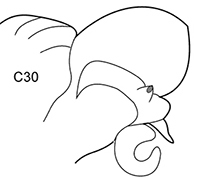	–	In middle chaetigers preacicular lobes rounded (rarely slightly bilobed in notopodia of very large worms). Branchiae without basal outgrows under dorsal cirri	**7**
	7(6)	Neuropodial postacicular lobes of middle chaetigers with distinct indentation on ventral side, distal to acicular lobes (arrow). Branchiae from C3 (rarely from C4; usually in small worms)	***N. longosetosa***
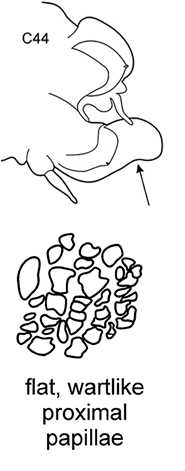	Body length up to 174 mm, up to 121 chaetigers. Branchiae from C3 to near posterior end. Preacicular lobes rounded, rudimentary, slightly bilobed in notopodia of very large worms. Acicular lobes of anterior chaetigers (and in middle chaetigers in large worms) bilobed. Postacicular lobes from C2 in neuropodia, C3 in notopodia. Notopodial postacicular lobes in anterior parapodia longer than acicular lobes, unequally bilobed with larger dorsal part; in middle and posterior parapodia shorter or slightly longer than acicular lobes. Neuropodial postacicular lobes of middle and posterior chaetigers much longer than acicular and notopodial lobes, with rounded tips and distinct indentation (arrow) on ventral side (best visible around C40). Spinose chaetae in postacicular rows. Pharynx with long middorsal subterminal papilla, up to 7 subterminal papillae per row, proximal region smooth or covered with flat warts in large specimens. Amphiboreal and Lusitanian, shelf depths, reported from Black Sea near Bosphorus (Rullier 1963), but these records require confirmation.		
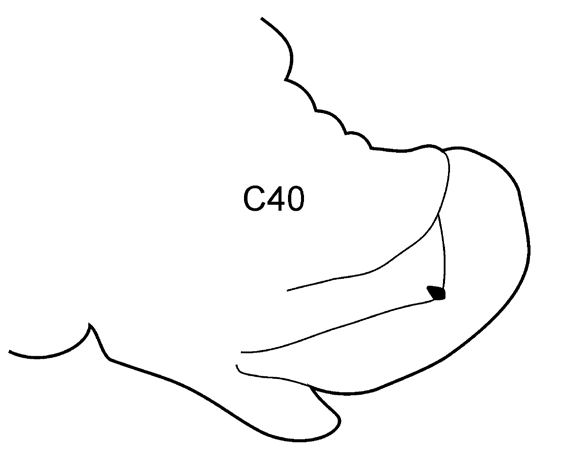	–	In middle chaetigers: no indentation on ventral side of neuropodial postacicular lobes (distal to acicular lobes). Branchiae always from C4 or later	**8**
	8(7)	Dorsal cirri in C1 poorly developed or even absent; notopodial cirri in posterior chaetigers as long as branchiae or longer; in middle chaetigers postacicular lobes distally longer (but not twice as long) than acicular lobes; aciculae lobes of noto- and neuropodia unequally bilobed (near C25–C35); proximal region of pharynx smooth; geniculate chaetae in postacicular rows	***N. cirrosa***
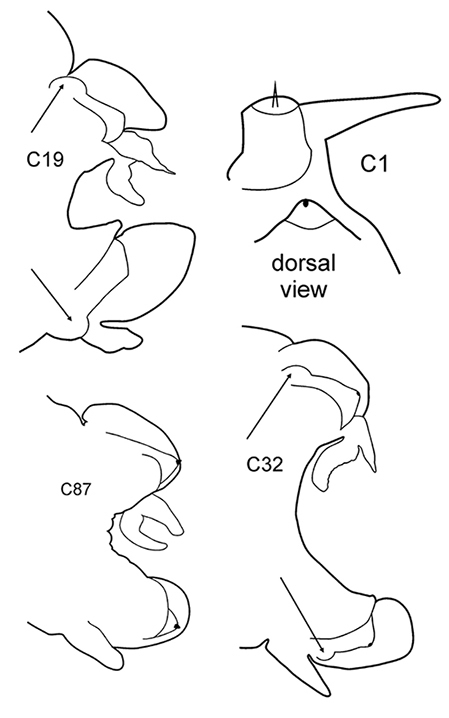	Body length up to 110 mm (Dnestrovskaya and Jirkov 2001), up to 100 chaetigers (Laborda 2004). Branchiae from C4 to near posterior end. Notopodial preacicular lobes rounded in anterior and middle chaetigers, rudimentary in posterior. Neuropodial preacicular lobes of anterior and middle chaetigers obliquely oval, distally not longer than acicular lobes, extending towards interramal region above acicular lobes; rounded in posterior chaetigers (behind C75). Notopodial acicular lobes broadly conical in anterior and conical in posterior chaetigers; unequally bilobed in C10–C45 with small round dorsal lobes and large round conical lobes with acicula (arrow). Neuropodial acicular lobes rounded or broadly conical in anterior and conical in posterior chaetigers; unequally bilobed in C10–C35 with small round ventral lobes and large round conical lobes with acicula (arrow). Postacicular lobes from C3 (rarely C2) in neuropodia, C3 in notopodia. All postacicular lobes distally longer (but not twice as long) than acicular lobes. Notopodial postacicular lobes rounded in anterior and posterior chaetigers, obliquely oval in middle chaetigers rounded posteriorly C70. Neuropodial postacicular lobes longer than corresponding notopodial postacicular lobes; rounded in most anterior chaetigers, oblique elongated with rounded tips in middle chaetigers (C10–C40), rounded after C60. Dorsal cirri in C1 absent or extremely small; in anterior and middle chaetigers conical with broad base; in posterior chaetigers digitiform, as long as branchiae or slightly longer; in 4–5 chaetigers preceding pygidium smaller than branchiae. Ventral cirri in C1 slender with slightly broad base, as long as prostomial palps, digitiform in posterior chaetigers. Up to 10–15 geniculate chaetae in postacicular rows (Marinov 1977); a peculiar trait of this species. Pharynx with slender middorsal subterminal papilla similar in size to largest subterminal papillae or slightly longer, 9–10 subterminal papillae per row (Rainer 1991; Hartmann-Schröder 1996) (up to 9 in holotype specimen). Proximal region of pharynx smooth. Low boreal and Lusitanian, upper shelf depths.		
	–	C1 with distinct dorsal cirri; notopodial cirri in all chaetigers half the length of the branchiae or even shorter; in middle chaetigers postacicular lobes more than twice as long as acicular lobes; aciculae lobes of noto-and neuropodia without any external outgrowths; proximal region of pharynx covered with small warts; geniculate chaetae absent	***N. caeca***
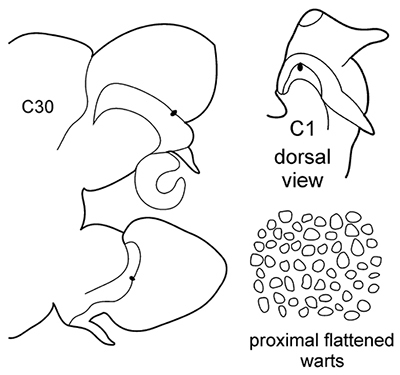	Body length up to 250 mm, up to 150 chaetigers (Rainer 1991). Parapodial preacicular lobes poorly developed rounded. Acicular lobe bilobed in anteriormost and middle regions of large worms. Postacicular lobes from C2 in neuropodia, C3 in notopodia, extending well beyond acicular lobes. Neuropodial postacicular lobes subequal in length to notopodial postacicular lobes or only slightly longer. Spinose chaetae in postacicular rows. Middorsal subterminal papilla of pharynx similar in size to largest subterminal papillae or absent; up to 6 subterminal papillae per row; proximal region covered with flattened warts. Amphiboreal and Lusitanian, reported from Black Sea near Bosphorus (Rullier 1963), but these records require confirmation; from the lower intertidal to nearly 1000 m (Rainer 1991), but according to our data it occurs at upper shelf depths.		
